# Flavonoids for treatment of Alzheimer's disease: An up to date review

**DOI:** 10.17179/excli2021-3492

**Published:** 2021-02-26

**Authors:** Jae Kwang Kim, Sang Un Park

**Affiliations:** 1 Division of Life Sciences and Bio-Resource and Environmental Center, Incheon National University, Incheon 22012, Korea; 2 Department of Crop Science and Department of Smart Agriculture Systems, Chungnam National University, 99 Daehak-ro, Yuseong-gu, Daejeon, 34134, Korea

## ⁯

***Dear Editor,***

Flavonoids, an omnipresent class of polyphenolic compounds, are commonly present in fruits, vegetables, and plant-derived beverages (Panche et al., 2016[36]). To date, more than 9000 structural variants of flavonoids have been identified (Williams et al., 2004[50]), most of which are important pigments that impart color to flowers to attract animal pollinators. Flavonoids protect against ultraviolet radiation, organisms that cause plant diseases, and herbivores. In addition, flavonoids play a role as physiological regulators, chemical messengers, and cell cycle inhibitors (Yonekura-Sakakibara et al., 2019[54]). 

Flavonoids are chemically composed of two aromatic ring systems (A and B rings) and a heterocyclic ring (C), forming a 15-carbon skeleton structure. This carbon structure can be abbreviated as C6-C3-C6 (Kumar and Pandey, 2013[26]). Based on the degree of unsaturation and substitution pattern, flavonoids can be divided into different subgroups, including anthocyanins, chalcone flavanols or catechins, flavanones, flavanonols, flavones, flavonols, and isoflavonoids (Santos-Buelga and Feliciano, 2017[38]).

Owing to the numerous inevitable biotic properties of flavonoids, they might act as anticancer, antioxidant, anti-inflammatory, antimicrobial, and antiviral agents. In addition, flavonoids have shown neuroprotective and cardioprotective effects in many clinical trials (Ullah et al., 2020[45]; Terahara, 2015[42]; Nijveldt et al., 2001[33]). Natural substances are considered to have robust protective effects against several unidentified diseases. Recently, it has been proven that they are most effective for the treatment of neurodegenerative diseases, including Alzheimer's disease (AD). Among the different natural compounds, flavonoids are used for their neuroprotective effects. In this review, we highlight the therapeutic potential of flavonoids, especially for AD. We report the current findings on the biological and pharmacological activities of flavonoids for the treatment of AD (Table 1(Tab. 1); References in Table 1: Ali et al., 2018[1]; Ano et al., 2019[2]; Bao et al., 2020[3]; Bortolotto et al., 2020[4]; Chakraborty et al., 2016[5]; Chen et al., 2020[6][7]; Currais et al., 2018[8]; Daily et al., 2020[9]; Diaz et al., 2019[10]; Dourado et al., 2020[11]; Guo, et al., 2017[12]; Hajizadeh Moghaddam et al., 2020[13]; He et al., 2018[14]; Heysieattalab and Sadeghi, 2020[15]; Hofmann et al., 2020[16]; Huang et al., 2018[17], 2019[18], 2020[19]; Ide et al., 2018[20]; Iida et al., 2015[21]; Inoue et al., 2019[22]; Jin et al., 2019[23]; Khan et al., 2019[24]; Kouhestani et al., 2018[25]; Li et al., 2019[27], 2020[28]; Lu et al., 2018[29]; Masood et al., 2020[30]; Meng et al., 2018[31]; Nakagawa and Ohta, 2019[32]; Oladapo et al., 2021[34]; Pacheco et al., 2018[35]; Ramezani et al., 2016[37]; Sohanaki et al., 2016[39]; Soleimani Asl et al., 2019[40]; Sonawane et al., 2019[41]; Thummayot et al., 2018[43]; Uddin and Kabir, 2019[44]; Wang et al., 2015[47], 2016[48], 2017[46]; Wei et al., 2019[49]; Xia et al., 2019[51]; Xian et al., 2012[52]; Yan et al., 2019[53]; Yu et al., 2015[56], 2019[55]; Zhao et al., 2019[57]; Zhong et al., 2019[58]; Zhou et al., 2020[59]; Zhu and Wang, 2015[60]).

| Key findings | Reference |
|---|---|
| **Flavones** | |
| Luteolin improves brain insulin resistance as well neuroinflammation, which might protect against the development of AD and the gut microbiota–liver–brain axis. | Daily et al., 2020 |
| Apigenin is considered an important neuroimmunomodulatory agent for the treatment of neurodegenerative conditions owing to its neuroprotective and anti-inflammatory effects. | Dourado et al., 2020 |
| Chrysin regulates hippocampal glutamate levels and Na+/K+-ATPase activity, which might play an important role in the reversal of memory deficit. | Bortolotto et al., 2020 |
| Baicalein dissolves preformed Tau oligomers as well as mature fibrils, suggesting its therapeutic potential for AD. | Sonawane et al., 2019 |
| Baicalin is a neuroprotective compound used for the treatment of microglia-mediated neuroinflammation during AD progression. | Jin et al., 2019 |
| Scutellarin exerts its beneficial effects on amyloid-β (Aβ)-related pathologies in the central nervous system by inhibiting the protein kinase B/nuclear factor-κB (NF-κB) signaling pathway. Further studies are needed to explore the efficiency of scutellarin in patients with AD. | Huang et al., 2019 |
| Hispidulin, a neuroprotective agent, protects against sevoflurane-induced neurological dysfunction and can improve the cognitive and memory function of elderly patients undergoing anesthesia. | Huang et al., 2018 |
| Wogonin could effectively increase amyloid-β (Aβ) protein clearance and decrease Tau phosphorylation, indicating its therapeutic potential against AD. | Zhu and Wang, 2015 |
| β-amyloid cleaving enzyme (BACE-1) is the main target for AD treatments. Acacetin decreases the production of human β-amyloid by transcriptional regulation of BACE-1 and amyloid precursor protein (APP), which results in the downregulation of APP protein expression and BACE-1 activity and consequently a decrease in the number of amyloid plaques. | Wang et al., 2015 |
| **Flavonols** | |
| Isoquercitrin protects hippocampal neurons from streptozotocin (STZ)-induced neurotoxicity, thus enhancing cognitive and behavioral impairment in STZ-induced AD rats. Hence, isoquercitrin is an effective therapeutic agent against STZ-induced neurotoxicity and AD-like changes. | Chen et al., 2020a |
| Troxerutin enhances the differentiation of neural stem cells (NSCs) and migration. It also neutralizes the inhibitory effects of Aβ42 on NSCs. Thus, it can be suggested that troxerutin is a potential lead structure to promote neurogenesis in neurological disorders such as AD. | Masood et al., 2020 |
| Quercetin was used for the development of an anti-AD formulation, which inhibited Aβ production *in vitro* and protected against cognitive impairments in a mouse model. | Nakagawa and Ohta, 2019 |
| Kaempferide has been reported to show neuroprotective effects. It decreased oxidative stress and improved the brain-derived neurotrophic factor (BDNF)/tropomyosin receptor kinase B (TrkB)/cAMP response element-binding (CREB) pathway in Aβ1-42-induced mice. | Yan et al., 2019 |
| Fisetin decreased cognitive deficits in old senescence-accelerated prone 8 mice while restoring multiple markers associated with decreased inflammation, stress, and synaptic function. These results indicate the therapeutic potential of fisetin against age-related neurodegenerative diseases. | Currais et al., 2018 |

EXCLI Journal 2021;20:495-502 – ISSN 1611-2156
Received: February 07, 2021, accepted: February 10, 2021, published: February 26, 2021

| Key findings | Reference |
|---|---|
| Kaempferol acts as an efficient neuroprotective agent against cognitive deficit in AD. Through elevating endogenous hippocampal antioxidants (superoxide dismutase and glutathione) and reducing neuroinflammation, kaempferol alleviated streptozotocin-induced memory damage in ovariectomized rats. | Kouhestani et al., 2018 |
| Myricetin treatment increased the number of hippocampal CA3 (cornu ammonis 3) pyramidal neurons and improved learning and memory damages in rats with AD. Thus, myricetin might be considered a beneficial compound for the treatment of AD. | Ramezani et al., 2016 |
| Rutin protects neuronal cells from amylin-induced neurotoxicity as well as oxidative stress. Thus, rutin administration could be a practical therapeutic approach to inhibit the development of AD, protect the aging brain, or slow down neurodegenerative processes. | Yu et al., 2015 |
| Isorhamnetin has been reported to protect against Aβ-induced cytotoxicity in human neuroblastoma SH-SY5Y cells. An *in vitro* Aβ aggregation trial test showed that isorhamnetin weakened Aβ fibrils. | Iida et al., 2015 |
| **Flavanones** | |
| Hesperetin (Hst) and nano-Hst have been used to effectively treat anxiety related to AD by upregulating the expression of cerebral antioxidant enzyme gene. | Hajizadeh Moghaddam et al., 2020 |
| In an *in vivo* study on an AD mouse model, sterubin played a potential role on both short- and long-term memory even at low dosages. | Hofmann et al., 2020 |
| Naringenin was used in an aging mouse model to evaluate the enhancement effect on cognition deficits in high-fat diet-fed SAMP8 mice. The possible mechanisms were elucidated by determining Aβ accumulation, oxidative stress Tau hyperphosphorylation, and neuroinflammation in the mice brain. | Zhou et al., 2020 |
| Naringin reduced the social-defeat stress-persuaded behavioral endophenotypes of neuropsychiatric disease by increasing glutamic acid decarboxylase-67 kDa synthesis through the inhibition of acetylcholinesterase (AChE) activity, neuroinflammatory processes in stress-sensitive brain regions, nitrergic stress, and oxidative stress. | Oladapo et al., 2021 |
| Sakuranetin showed a protective effect on brain cells via an antioxidant mechanism. Additionally, the effectiveness of sakuranetin in learning and memory damages might be associated with the inhibition of inflammatory mediators in brain tissues. | Li et al., 2019 |
| Eriodictyol improves lipopolysaccharide (LPS)-induced amyloidogenesis and memory damage via preventing toll-like receptor 4 (TLR4), mitogen-activated protein kinases (MAPKs), and phosphatidylinositol 3-kinase (PI3K)/protein kinase B (Akt) and activating sirtuin 1 (SIRT1) pathway, thus blocking the downstream translocation of NF-κB. This indicates a potential therapeutic approach for AD. | He et al., 2018 |
| Pinocembrin showed a positive protective effect against Aβ25-35-induced neurotoxicity in SH-SY5Y cells via activating the nuclear factor erythroid 2-related factor 2 (Nrf2)/heme oxygenase-1 (HO-1) pathway and inhibiting mitochondria-dependent apoptosis. These mechanisms help protect cells from Aβ25-35-induced neurotoxicity. | Wang et al., 2016 |
| Hesperidin shows moderate 2,2'-azino-bis (3-ethylbenzothiazoline-6-sulfonic acid) (ABTS) positive radical scavenging activity. However, DNA nicking assay revealed strong hydroxyl radical scavenging ability. This finding validates the importance of a novel multi-target screening process to identify multi-potent agents from natural source for AD therapeutics. | Chakraborty et al., 2016 |
| Pinostrobin exerted neuroprotective effect against Aβ(25-35)-induced neurotoxicity in pheochromocytoma cells via inhibiting oxidative damage, calcium overload, and the mitochondrial pathway of cellular apoptosis. | Xian et al., 2012 |

EXCLI Journal 2021;20:495-502 – ISSN 1611-2156
Received: February 07, 2021, accepted: February 10, 2021, published: February 26, 2021

| Key findings | Reference |
|---|---|
| **Flavanonols** | |
| Engeletin decreased Aβ1-42-induced oxidative stress and inflammation in BV-2 cells by regulating the Keap1/Nrf2 pathway. These findings indicate the potential of engeletin as an AD therapeutic. | Huang et al., 2020 |
| Isoastilbin has shown to be effective for AD owing to its antioxidant and anti-apoptotic properties. | Yu et al., 2019 |
| Taxifolin showed intracerebral pleiotropic neuroprotective effects on cerebral amyloid angiopathy (CAA) by reducing Aβ production and modulating proinflammatory microglial phenotypes. | Inoue et al., 2019 |
| Astilbin exerts positive effects such as lessening learning and memory deficits and reducing plaque burden and Aβ levels. In the astilbin-treated group, the expression levels of CREB protein as well as brain-derived neurotrophic factor (BDNF) were significantly upregulated. In addition, the disturbance of AKT/GSK-3β signaling pathway was markedly enriched in the hippocampus (Hp). These findings recommend that astilbin could be a potent therapeutic agent against AD. | Wang et al., 2017 |
| **Flavanols or Catechins** | |
| Epigallocatechin-3-gallate (EGCG) reduces AD-like cognitive damages through its anti-amyloidogenic, anti-inflammatory, and neuroprotective effects. Therefore, it might be a promising therapeutic candidate for AD. | Bao et al., 2020 |
| (-)-epigallocatechin and (-)-epicatechin-3-gallate (ECG) have been shown to reduce the toxicity of Aβ oligomers and fibrils. ECG passes through the blood–brain barrier to reduce brain Aβ plaques in APP/PS1 mice, thus protecting neurons from damage. These results show the effectiveness of (-)-epigallocatechin and ECG in alleviating the symptoms of AD. | Chen et al., 2020b |
| Procyanidins are used to reduce the pathological features of AD, extracellular amyloid deposits, and neurofibrillary tangles via inhibiting Aβ accumulation and Tau pathology. The improvement of cognition as well as variation of synaptic plasticity by these compounds also contributed to the alleviation of AD. | Zhao et al., 2019 |
| Aflavins can inhibit neural inflammation and protect from AD and depression-related disorders, which are mainly caused by inflammation in the brain. | Ano et al., 2019 |
| Epicatechin reduces Aβ25-35-induced neurotoxicity, immunoreactivity of heat shock proteins (HSP)-60, -70, and -90, and neuronic death in the CA1 (Cornu Ammonis 1) region of the Hp of rats injected with Aβ25-35. These changes are considered to enhance the function of spatial memory. | Diaz et al., 2019 |
| Epigallocatechin-3-gallate attenuates microglial inflammation and neurotoxicity through inhibition of both canonical nucleotide oligomerization domain-like receptor pyrin domain-containing protein 3 (NLRP3) and noncanonical caspase-11-dependent inflammasome activation via the TLR4/NF-κB pathway. | Zhong et al., 2019 |
| Catechins have both antioxidant and anti-inflammatory effects. The potential effects of these compounds in AD prevention and regulation have been reported in *in vitro* and *in vivo* studies. | Ide et al., 2018 |
| (-)-Epigallocatechin-3-gallate consumption reduced impairments in spatial learning and memory and decreased the reduction in synaptic proteins in an AD mouse model. Thus, EGCG could be a novel candidate against neurodegenerative diseases. | Guo, et al., 2017 |

EXCLI Journal 2021;20:495-502 – ISSN 1611-2156
Received: February 07, 2021, accepted: February 10, 2021, published: February 26, 2021

| Key findings | Reference |
|---|---|
| **Anthocyanins** | |
| Anthocyanin consumption improves AD-induced cognitive dysfunction. In addition, it protects against hippocampal neuroinflammatory responses and induces the phagocytosis of microglia to Aβ protein plaques, downregulates inflammatory factors (CD33), and upregulates microglia homeostatic factors [triggering receptor expressed on myeloid cells 2 (TREM2) and TYRO protein tyrosine-binding protein (TYROBP)] by regulating the CD33/TREM2/TYROBP signaling pathway in microglia. | Li et al., 2020 |
| Delphinidin is a plate-like molecule intercalated between β-plated sheets related to Aβ molecules, and it repressed the formation of amyloid fibrils. Thus, it might be a potential therapeutic agent against AD and other related cognitive disorders. | Heysieattalab and Sadeghi, 2020 |
| Anthocyanins could be a safe healing agent for reducing inflammation-induced neurodegeneration in the brain in several diseases, especially AD and Parkinson's disease (PD). Several pathological studies have shown amelioration of these diseases in LPS-induced animal models following treatment with anthocyanins. | Khan et al., 2019 |
| Amyloid β enhanced escape latency and distance traveled in the Morris water maze task. Pelargonidin reduced these behavioral changes. Aβ decreased the total thiol content of the Hp, and pelargonidin restored the hippocampal antioxidant capacity. | Soleimani Asl et al., 2019 |
| Cyanidin reduced Aβ-induced inflammation and ROS production via the TLR4/NOX4 pathway, suggesting that inhibition of TLR4 by cyanidin might be effective in avoiding neuronal cell death in AD. | Thummayot et al., 2018 |
| Anthocyanins protected SH-SY5Y cells against Aβ1-42-induced apoptosis by regulating apoptosis- and Ca2+ homeostasis-related genes and preventing mitochondrial dysfunction. | Meng et al., 2018 |
| Anthocyanins reduced memory deficits, protected the brain from oxidative damage, and restored AChE and ion pump activity in an STZ-induced sporadic dementia of Alzheimer's type rat model. | Pacheco et al., 2018 |
| Anthocyanins serve as effective antioxidant neuroprotective agents against amyloid-beta oligomer (AβO)-induced neurotoxicity in HT22 cells via PI3K/Akt/Nrf2 signaling. Notably, anthocyanins restored memory-related pre- and postsynaptic protein markers and memory functions in amyloid precursor protein/presenilin-1 (APP/PS1) mice. | Ali et al., 2018 |
| Pelargonidin restored Aβ25-35-induced memory deficit by reducing oxidative stress, cholinergic dysfunction, and astrocyte reaction. | Sohanaki et al., 2016 |
| **Isoflavonoids** | |
| Genistein protected against Aβ protein-induced cognitive impairments and exerted antioxidant properties to scavenge AD-mediated generation of free radicals. In addition, genistein interacts directly with the targeted signaling proteins and stabilizes their activity to combat AD. | Uddin and Kabir, 2019 |
| Daidzein shows significant improvement in intracerebroventricular-streptozotocin (ICV-STZ)-induced memory and learning impairments. It was proven using the Morris water maze test and spontaneous locomotor activity. | Wei et al., 2019 |
| Sophotokin is considered a new pterocarpan-type anti-inflammatory compound against neuroinflammation-related diseases. The anti-neuroinflammatory mechanism involves the inhibition of TLR4 signal pathway at the sites of NF-κB and MAPK with PU.1 as a likely upstream target. | Xia et al., 2019 |
| Soy isoflavones show neuroprotective effects on cognitive dysfunction induced by scopolamine, indicating that they might be suitable candidates for neurodegenerative diseases, such as AD. | Lu et al., 2018 |

*Acknowledgments*

*Conflict of interest*

The authors declare no conflict of interest.

## Acknowledgements

This research was supported by the Bio & Medical Technology Development Program of the National Research Foundation (NRF) funded by the Ministry of Science, ICT & Future Planning (2016M3A9A5919548).

## Conflict of interest

The authors declare no conflict of interest.

## Figures and Tables

**Table 1:**
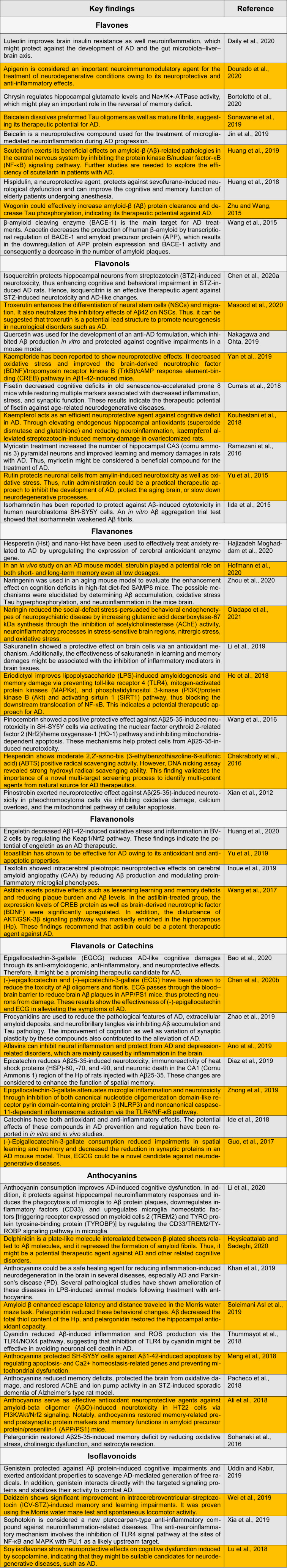
Pharmacological and biochemical activities of flavonoids for the treatment of Alzheimer's disease reported recently
